# Towards institutionalised regionalism: the role of institutions and prospects for institutionalisation in ASEAN

**DOI:** 10.1186/2193-1801-3-556

**Published:** 2014-09-24

**Authors:** Pattharapong Rattanasevee

**Affiliations:** Department of Social and Policy Sciences, University of Bath, Claverton Down, Bath, BA2 7AY UK

**Keywords:** ASEAN, Institution, Institutionalisation, Regionalism, Southeast Asia

## Abstract

This paper provides concrete understanding of the role of institutions and prospects for institutionalisation in ASEAN. It highlights the significant roles of institutions in the integrating Southeast Asia and demonstrates three main areas of institutional deficiency in the association. However, although ASEAN institutions meet the expectations, by and large, in terms of serving the national governments and upholding the association’s norms, along with theoretical explanation, this paper argues that ASEAN should focus on strengthening its existing institutional structure by providing them with a mandate and sufficient financial and human resources in order to support its administration and growing activities. Finally, the paper suggests a revision of the current financial contribution system, a seeking of alternative sources of income and some institutional inventions such as a research wing and a mechanism that can get non-state actors involved in the process.

## Introduction

Despite regional integration having been introduced in Europe since 1950s, in Southeast Asia it was not until the 1980s that it began to gain attention. The 1997 financial crisis highlighted the high degree of interdependence among ASEAN members and the urgent need for closer cooperation towards more intensive regional community building. To fulfil this ambition and match the speed of European integration, institutional development is widely thought to be one of the most important driving factors. In fact, ASEAN has undergone significant transformation over recent decades by revamping its institutional structure in ways that can now support its administration and activities in such a way as to be able to invite comparisons with the European Union (Jetschke and Murray 2011: 176). Such efforts can be found for example in the ASEAN Vision 2020 introduced in 1997, the 2003 Bali Summit and the 2004 Vientiane Action Programme. More recently, the ASEAN Charter adopted in 2008 appears to be the most important agreement aimed at strengthening its institutional organs. That is, the Charter has improved the organisation’s implementation and dispute-settlement mechanisms, consolidated its decision-making structure and provided the association with more rule-based conditions as well as more legally binding obligations. Nevertheless, its weak institutional structure and a significant gap between its rhetorical goals of cooperation and its actual achievements have always been a mainstream criticism of ASEAN and demonstrate an area of great deficiency. This problem greatly limits ASEAN’s capacity to enhance an integration process and adopt a genuinely regional approach to tackle transnational problems. Although ASEAN leaders realize this weakness and continue to strengthen the institutional mechanisms, it is understood that those commitments are clearly insufficient and ineffective due to being constrained by the principles of ASEAN Way and other important factors that continue to limit the role of ASEAN institutions.

To reiterate the role of institutions, new institutionalism claims that strong institutions are a prerequisite for constructing a regional community, for they act as the rules of the game and they link all actors and their action together. Similarly, integration theories also highlight the importance of institutions in the process. Under neofunctionalism, institutions are viewed as the backbone of the association where all political activities take place, while liberal intergovernmentalism underlines their role in facilitating and accelerating the integration process to achieve mutually desired outcomes, for example, by providing reliable information, monitoring compliance and linking across issues. However, in the context of ASEAN where the member states are highly influenced by the normative underpinnings of the ASEAN Way, a better understanding can be gained from constructivism as it appears to be a more comprehensive framework to explain international behavior and highlight the relationships between rules, norms, institutions and identities. Under the constructivist lens, regional integration is depicted as a social interaction, which includes international contexts by considering states and institutions as cognitive and correlative entities. This supports the perspective that international contexts are vital for understanding institutions in the regional settings as they construct states’ identities and interests. In sum, these theories identify the key roles of institution and hold that to construct a successful regional community a certain form of institutional arrangements is required.

In terms of institution, the case of ASEAN could make an insightful contribution to the study of regional integration. It contrasts the picture of European integration in that so far it has mainly bypassed the role of the institution, concentrating more on the influence of national governments in the process. Consequently, examining the institution in the context of ASEAN and its impact on integration would shed new light on regional contexts in the developing world that do not have a tradition of strong institutions. In this paper, I will begin with providing a concise background of institutional development in ASEAN and the current institutional structure of the association after being enhanced in 2007 by the ASEAN Charter. Then, I move on to discuss concepts about institutions from the viewpoint of constructivism, as a main theoretical approach in this study. The following section provides empirical evidence for evaluating the role of institutions in the development of ASEAN’s integration. The final section concludes this study by providing further discussion and a summary of the important findings as well as giving constructive suggestions for solutions to the problems identified. This paper uses documents and interviews with technocrats and scholars as the main research methods. This research is undertaken in accordance with the University of Bath Ethical Guidelines and Code of Good Practice in Research.

## Background

From the outset, the development of ASEAN institutions was highly influenced by a set of ideas that were shaped by the regional cultural values and events that ‘preoccupied politicians, intellectuals and opinion leaders over many years’ (Stubbs 2008: 455). Regarding this, the history, ideas and the distinctive traits of ASEAN Way have served to define an appropriate means of diplomacy and multilateralism, which could provide an explanation for the nature of institutionalisation within the region. In relation to history, it can be traced back to the timing of the formation of ASEAN in 1967. The end of confrontation between Indonesia and Malaysia, the rise of nationalism resulting from colonial experiences, the gaining of independence by ASEAN members, the spread of communism and the Cold War that took a strong hold on the region were the key drivers that helped to ‘shape the ideational basis of the association and its initial trajectory’ (Stubbs 2008: 456). Similarly, Narine (1998: 33) pointed out that there were interrelated objectives involved in the creation of ASEAN: ‘to alleviate intra-ASEAN tensions, to reduce the regional influence of external actors and to promote the socioeconomic development of its member states as a further hedge against Communist insurgency’. These concerns continued to influence the association until the end of 1970s, when the member states started to prosper, mainly from increasing foreign direct investment, and gain more confidence, which resulted in several treaties and declarations as well as institutional initiatives. To some extent, this affirms the belief that the initial decision and intention to establish ASEAN were productive and hence had paid off.

ASEAN is well-known for its informality, personalism as well as reluctance and discomfort towards the adoption of solid formal institutions and legally binding obligations. The internal dynamics of ASEAN institutions has been designed based on the principal values of the organisation. Accordingly, the avoidance of armed conflict, the non-interference in the internal affairs of other states, the consensus-based decision making and the non-legally binding agreements give plenty of space for member states to exercise their rights and powers freely as well as to pursue their regional agendas. To some extent, this helps to create a scene of equality in that the smaller member states feel comfortable when dealing with larger partners. The ASEAN’s norms are a strong commitment to the idea of state sovereignty which was ‘the most important protection against the internal and external weaknesses of the ASEAN states’ (Busse 1999: 46). Collins (2007: 212) posited that ‘ASEAN was therefore established to ensure sovereignty remained firmly located at the national level’ and clearly not intended to be a supranational institution, equivalent to the European Union. However, as a consequence of its members unwillingness to delegate powers to the institutional bodies, ASEAN lacks effective institutional mechanisms to support, pursue and deliver its objectives and policy initiatives as well as being unable to act against the will of a member state. With the absence of a strong central authority, policy delivery and forceful compliance mechanisms, ‘ASEAN continues to rely primarily on the collective will of its member states, their perceived national interests, and peer pressure to ensure compliance with its agreements and decisions’ (Asian Development Bank 2010: 125). This situation has resulted in a poor record of implementation and a number of well-voiced criticisms. For instance, ASEAN is often referred to as “a talk shop”, “a toothless tiger” or “big on words but small on action”.

### ASEAN’s institutional structure after the implementation of the ASEAN Charter

**Figure 1:**
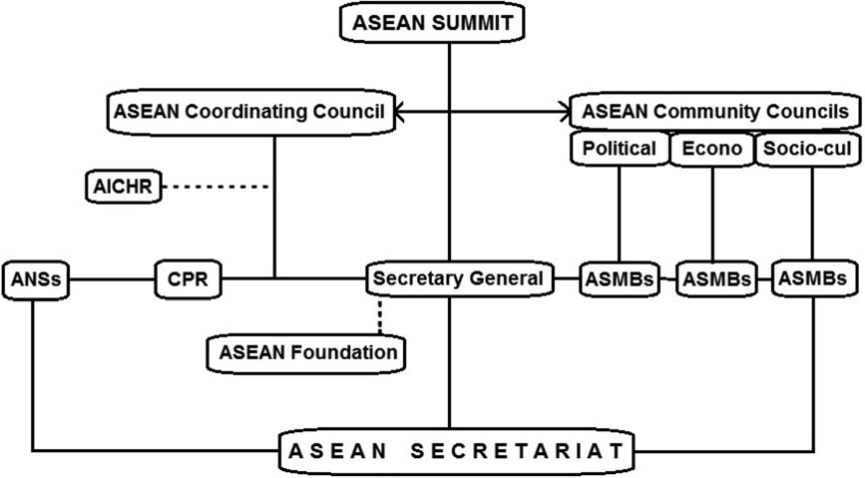
**ASEAN’s institutional organs after the ASEAN Charter.**

In terms of institutionalisation, the most significant step forward was taken in 2007. The ASEAN Charter serves to provide legally binding and institutional framework for accomplishing the ASEAN community. It sets out rules, norms, values, budget and finance, administrative procedures as well as the structure of institutional bodies. As can be seen in Figure 1, the institutional structure of ASEAN is relatively complex and has wide horizontal configuration. As described in the ASEAN Charter,^1^ the organisation has nine main institutional bodies as follows. The **ASEAN Summit** is the supreme institutional organ of ASEAN, taking the form of an annual meeting comprising the heads of member states and dialogue partners and is responsible for ‘taking decisions on key issues pertaining to the realisation of the objectives of ASEAN, important matters of interest to member states.’ The **ASEAN Coordinating Council (ACC)** attended by the foreign ministers of the member states who meet at least twice a year, is responsible for preparing the summit and coordinating ‘the implementation of agreements and decisions of the ASEAN Summit’ and with the ASEAN Community Councils is tasked with enhancing policy coherence, efficiency and cooperation across these institutions. The **ASEAN Community Councils** together comprise the Three Pillars of ASEAN Community Councils the: ASEAN Political-Security Community Council, ASEAN Economic Community Council, and the ASEAN Socio-Cultural Community Council. The objectives of each pillar are to coordinate the work of the relevant sectors, ensure the implementation of the relevant decisions as well as to submit reports and recommendations to the summit. The **ASEAN Sectoral Ministerial Bodies** are in charge of implementing the agreements and decisions of the ASEAN Summit, strengthening cooperation in the field and submitting reports and recommendations to their respective Community Councils.

**The Secretary General of ASEAN and the ASEAN Secretariat** form the organisation’s central administrative body. These staff work only for ASEAN and should not seek or receive instructions from any government or external party. The Secretary General is appointed by the ASEAN Summit for a non-renewable term of five-years and is assisted by four Deputy Secretaries-General who come from four different ASEAN member states. The **Committee of Permanent Representatives to ASEAN (CPR)** is a new important body, formerly known as the Standing Committee, which consist Permanent Representatives to ASEAN who act as its ambassadors. They ‘support the work of the ASEAN Community Councils and ASEAN Sectoral Ministerial Bodies’ as well as being tasked to ‘liaise with the Secretary-General of ASEAN and the ASEAN Secretariat on all subjects relevant to its work and facilitate ASEAN cooperation with external partners.’ The **ASEAN National Secretariats** serve as ‘the national focal points, being the repository of information on all ASEAN matters at the national level, coordinating the implementation of ASEAN decisions at the national level.’ The **ASEAN Intergovernmental Commission on Human Rights (AICHR)**, created to fulfil the objectives of promoting and protecting human rights and fundamental freedoms, operates ‘in accordance with the terms of reference to be determined by the ASEAN Foreign Ministers Meeting.’ Finally, the **ASEAN Foundation** was established to ‘promoting greater awareness of the ASEAN identity, people-to-people interaction, and close collaboration among the business sector, civil society, academia and other stakeholders in ASEAN.’ That is, it supports the Secretary-General of ASEAN and cooperates with the relevant institutions to promote ASEAN community building. Moreover, the Charter has paved the way for ASEAN to engage with other entities that support its purposes and principles, which can include organisations in the areas of: business, academia, civil society and science and technology.

Overall, the introduction of the ASEAN Charter was a significant progress, which was aimed at promoting institutional development and silencing growing criticism by providing ASEAN with more rules-based and legally binding foundations, Moreover, it had the goal of the creation of the ASEAN Economic Community (AEC) as a single market and production base by 2015. It has been attested that the Charter ‘improves the organization’s compliance mechanisms, streamlines its decision-making structure, and extends its dispute-settlement mechanism’ (Asian Development Bank 2010: 124). The ASEAN Secretariat and the Secretary General can be construed as the core administrative mechanism of ASEAN’s institutions and is provided with its own financial resources and professional staff. However, the Charter did not change the Association’s adherence to the principle of non-intervention into domestic affairs, the dominant emphasis on national interests and the manner of elitism (Jetschke 2009:418). As a lone central authority and the centre of institutional dynamics, the ASEAN Secretariat, though having many important responsibilities, still has very limited power. Before the restructuring of ASEAN institutions in 1992, it served as a channel of information and was never meant to be an important body that would be able to command ASEAN activities and set agendas. Despite it having been accorded ministerial status and assigned to monitor and implement ASEAN policies after the Fourth ASEAN Summit in 1992, the Secretariat still has little input on policy initiatives, only operating in accordance with the directives issued by the ASEAN leaders and foreign ministers (Hernandez 2007: 11). Even with the recent improvements in mandate and monitoring compliance given by the Charter, ASEAN is still a very top-down organisation, with the Secretariat still having to face many difficulties, as although they have to implement the policy of its leaders, they lack the resources, in terms of funding and qualified staff, to perform their function. Regarding this, Suryodiningrat (2009) has argued that ‘in practice, the secretary-general remains an official prostrate to the member countries’. Thus, it is apparent that ASEAN institutions would continue to have limited power for effectively policy implementation as long as there has not been a significant shift in the vision of the organisation’s leaders. In particular, the principles of sovereignty and non-interference, which have been rigidly upheld by its member states, need to be revisited.^2^

### Constructivism and ASEAN institutions

Although new institutionalism precisely provides the means to think theoretically about institutions as well as their roles and their influences on behaviour and outcomes, it does not exemplify the dynamics or interactions with other important elements in the process. Furthermore, it does not give a clue about how to set up or design an appropriate arrangement of institutions based on the context of regional settings. On the other hand, constructivism takes a sociological concept of action to extend the scope of theoretical explanation by focusing on the importance of institutions to state action in relation to norms and by bringing the international context into the discussion. It argues that human action and behaviour are driven by norms, rules, institutions and identities. Under this lens, rules and institutions are seen as products made by human practices and states as well as institutions are viewed as cognitive and correlative entities. Moreover, the outcomes are shaped by the current institutions and also by actors’ learning through the previous ones. More importantly, according to Narine (1998), almost all international relationships that shape states’ identities and interests are developed within an institutional context and institutions themselves act as representatives of the rules and norms of these international interactions. In other words, with this perspective institutions are tasked with securing and maintaining the norms as well as the constructed identities. Moreover, institutions in the constructivist’s view are not rigid as argued by new institutionalist scholars. Accordingly, they, including interests and identities, can be altered by the integration process at the systematic level through various kinds of interactions (Wendt 1992, Wendt 1995). This introduces the idea that the surrounding environments, external constraints and international contexts are vital for understanding institutions in the regional settings. Thus, according to Cini (2007: 131), institutions are ‘arenas for communication, deliberation, argumentation, persuasion and socialization’ among actors.

In the context of European integration, the role of institutions has clearly accorded with most of the theoretical explanations in that they are at the heart of the integration process. That is, they play a key role in initiating and setting the agenda, law-making, budgeting, coordinating, implementing policies as well as monitoring procedures. They are the backbone of the union and a place where member states pool some of their sovereignty, resources and confidence. All these functions are legally underpinned as prescribed in the EU Treaties and ratified not only at the central level but also by members’ parliaments, which thus provide robust legitimate power for its institutions to perform EU’s activities. However, although EU institutions are solid, powerful and seen as a principal feature of European integration, to a certain extent, national governments still play a pivotal role in determining what policy is made. In sum, the evidence regarding the EU concurs with the view that the institutional configuration required in a regional integration project is determined by the ‘historical circumstances of the group of countries’ (Best 2005: 43).

Unlike integration theories and new institutionalism, constructivism is capable to explain the role of norms and collective identity in the context of ASEAN integration. As the ASEAN Way evidently plays a vital part in the dynamics of ASEAN integration, hence constructivism can deliberately explain the formation of collective identity at regional level and could potentially be one influential approach to analyse subjective issues or things relating to the ideational force within integrating regions. Acharya (2005: 95) asserted that constructivism offers ‘a more complete explanation of ASEAN’s achievements and failures’ in which international action and institutional-building are seen as ‘shapers of the regional balance of power’. Similarly, Busse (1999: 39–41) viewed that we can gain a better understanding from constructivism because ASEAN members, to some extent, have moved away from pure balance-of-power politics towards establishing a regional code of conduct which rotates around norms. He concluded that such adherence to norms has become an essential part of their foreign policy and must be seen as a basis of their national interests. On the whole, due to the low degree of institutionalisation, the non-legalistic approach to cooperation and limited resource capacity, I am of the view that, for ASEAN, the normative underpinnings and ideational force have more play in solidifying and maintaining the association. Thus, viewing ASEAN through the lens of constructivism is indispensable and no serious study of ASEAN could be completed without careful consideration of the role of norms.

### Re-emphasising the problems of institutional deficiency: an empirical investigation

In the context of ASEAN, its institutions have always been at the centre of criticism about the association. Moreover, according to the collected research data, institutions have been a problematic area and given their importance as purported regional integration theory, clearly require attention. Repeatedly, ASEAN leaders and many observers have accepted that the problem of institutions is a major weakness which has resulted in a number of attempts in strengthening the ASEAN Secretariat and other institutional bodies recently, most explicitly seen in the ASEAN Charter. However, it is argued that those efforts have been insufficient to make them a reality owing to several obstacles that continue to limit the role of its institutions. First of all, any study of ASEAN institutions would uncover that ASEAN fundamentally has to work within the context of intergovernmentalism, whereby the organisation prefers non-intrusive decision-making and has no intention of following a path of supranationalism. That is, it has been ‘deliberately designed for flexibility to allow national governments sufficient autonomy in deciding which sectors to liberalise deregulate or reform and at what speed’ (Nesadurai 2012: 4). Apparently, perhaps as reflected by its name, the ASEAN Secretariat is never far away from being a glorified secretary who works in an office sorting out the daily paper work, making phone calls and arranging meetings for the organisation, and never given the power to make important decisions and initiate policy. This perspective was backed by an Indonesian scholar from CSIS who depicted the ASEAN Secretariat as:

*“The ASEAN Secretariat is exactly like the name. It is a secretariat. It is for administrative issues only… documents, letters. It is not a decision-making body.”*^*3*^

The first matter that I will address is regarding the role of the ASEAN Secretariat and its performance. As discussed previously, the ASEAN Secretariat and the Secretary General of ASEAN, although having been enhanced by the Charter, still have a very limited role in policy-making and are not capable of acting against obstinate members. There is quite a significant number of evidence supporting this view. For example, Hund (2002: 118) contended that ‘the ASEAN Secretariat remains at the margins of ASEAN policy-making’ as it does not possess delegated powers for commanding individual member state compliance or devising common policies on its own initiative. Similarly, according to Capannelli and Tan (2012: 14), the ASEAN Secretariat’s remit has principally been to ‘furnish administrative support rather than been invested with powers of delegation… and did not aim to create regional bureaucracies promoting a more independent agenda for integration beyond the scope provided by intergovernmental cooperation structures’. In addition, a recent review conducted by Desker (2008) found that during the preceding forty years only 30% of ASEAN agreements and initiatives were actually implemented, which points to the incapacity of the ASEAN Secretariat and he concluded that it does need to be strengthened. Nevertheless, as advised by constructivism, I personally believe that ASEAN leaders, whose action and decision are considerably constrained by the norms, still wish to maintain the national autonomy, its policy initiation and supreme decision making. Therefore, in order to coordinate ASEAN activities and avoid conflicts of national interests, I am convinced that expansion in the size and strengthening of the implementation and compliance mandate of the ASEAN Secretariat are required. This would help improve the association’s efficiency, the record of agreement implementation and, in one way, speed up the process of ASEAN integration. As pointed out by Beeson (2008: 34), ‘what is of potentially greatest importance, however, is the commitment to effective implementation as well as compliance with ASEAN agreements’.

Strengthening the ASEAN Secretariat prompts concerns about how this could be funded. In general, it is widely agreed that the Secretariat has inadequate financial and human resources to manage the association’s growing activities and to service the needs of deeper regional cooperation. Its operational budget of the ASEAN Secretariat mainly relies on equal annual contributions by the member states, thus reflecting the norm of equality across the organisation and stemmed from the belief that different contributions might lead to a hierarchy of powers. It has been pointed out that ASEAN’s system of equal contributions is unique among international organisations that its leaders have avoided any substantial increase and held back to the level of the poorest members’ capacity to pay (Severino 2009: 25). In other words, the budget is kept low enough for the poorest state to be able to pay without being too demanding on its resources. However, as a lone central institution, the Secretariat is overloaded with region-wide administrative and coordinative activities as well as research, analysis, technical support and monitoring tasks (Nesadurai 2012: 16). Although ASEAN does not make its financial statement available for the public, Termsak Chalermpalanupap, a former Director for Research and Special Assistant to the Secretary-General of ASEAN, wrote that ‘in the 2007–08 financial year, the Secretariat’s operating budget was US$ 9,050,000’ (Chalermpalanupap 2009: 122). Additionally, he also noted during interview:

*“Our budget this year (2012–2013) is only US$ 15.763 million and next year I heard there is only going to be a 3% increase. So, it is still a very small shoestring operation.”*^*4*^

If the 2012–2013 figures are taken into consideration, this means each member contributed US$ 1,576,300 and represents a very small proportion of their GDPs or annual national budgets. For instance, according to Bower (2010) and Poole (2011: 6–7), the total budget of the ASEAN Secretariat was reported to be around US$14.3 million (including funds from partner countries), which accounted for 0.0001% of Laos’s GDP and 0.000001% of that of Indonesia and amounts to about 0.137% of the EU’s annual administrative budget. Calculated in terms of per capita, ASEAN citizens spend less than $0.024 per person per year on supporting the servicing of the organisation.^5^

At the same time, ASEAN members, of course, contribute to the association in some other ways, for example, the offices of the National Secretariat are housed in members’ foreign ministries. Some countries run special projects as well as hosting and attending meetings or events. Furthermore, ASEAN is also substantially funded by dialogue partners or external donors, mostly through specific projects or operations, such as capacity building, improving infrastructure and information technology. Interestingly, Bower (2010) noted that the external contributions are well over 20 times the Secretariat budget. The former Secretary-General of ASEAN reinforced this point when he stated at interview:

*“We have many cooperation projects with different dialogue partner countries. These countries are developed nations. They are willing to give more resources to ASEAN. We always say ASEAN is an OPM organisation – Other People’s Money.”*^*6*^

However, relying on external resources could lead to unavoidable external interference by the donors in the affairs of the association. Certainly, this is not sustainable in the long run and not sensible if ASEAN wants to present itself as a non-aligned and independent power on the international political stage. Another important matter is with regards to the lack of professional staff. According to the figures, in 2012 ASEAN employed roughly 300 people, including 65 managers and experts, 180 local staff and 55 persons from donor organizations (ibid). Although these figures do not include coordinating staff who work in member countries, they are minuscule compared to the EU, which has about 55,000 staff working under the umbrella of its institutions.^7^ Nevertheless, there are some detractors from the view that ASEAN is underfunded, who prefer to highlight the problem of staff shortages. For instance, the former Secretary General contradicted the previous arguments and pointed out that the main problem of the ASEAN Secretariat is not lacking budget, but rather the lack of well-trained bureaucrats. He shared:

*“It’s always being said there is not enough budget. But in the last few years, my understanding is that the budget is not spent… The problem is that we are lacking in capacity. We don’t have enough ASEAN-level bureaucrats working at the Secretariat to make sure that all the implementation is carried out.”*^*8*^

This view has been supported by Severino (2009: 25), who wrote ‘the problem is not only the availability of funds. It is also the difficulty of finding personnel who know ASEAN and the requirements and limits of regional, especially economic, cooperation and integration’. Similarly, this coincides with the viewpoint of Indonesia’s Permanent Representative to ASEAN. He made the following comment:

*“Do you know that every year the Secretariat also has to return the money from the contributions of the governments? There are some unspent budgets. (Why the budget is not spent?) Many reasons, improved efficiency, lack of staff… We have to differentiate between operational budget and activity budget. Operational budget based on the contribution of the governments and every year we have unspent budget. For activities, I think we need more. We don’t have enough.”*^*9*^

As has been seen, despite the insufficiency of staff, the ASEAN Secretariat is also facing difficulties in efficiency and in attracting talented and capable people. Areethamsirikul (2010) pointed out that ‘the ASEAN headquarters needs to create a regional and international working atmosphere and to make "working at ASEAN" a prestigious assignment for ASEAN peoples - akin to the perception of working at the United Nations, the World Trade Organisation, the World Bank, or the European Union.’ Thus, the budget is not the only important concern in this respect and I observe that as long as working for the Secretariat is not perceived as a well-paid and challenging career as well as cannot attract ambitious, capable and professional talent, it will be difficult for the ASEAN Secretariat to grow into a more institutionalised organ, a powerful central administration and/or the backbone of the association.

Finally, considering the sophisticated structure of the EU’s institutions, this raises the question whether ASEAN should follow suit. In other words, what should be improved for ASEAN in terms of institutional development? Although constructivism and other theories imply that institutions play a vital role in the development of regional integration, the question is whether it is appropriate to “copy and paste” one successful model to another context. In general, there are quite a variety of opinions and views on this issue. However, the majority of evidence suggests that rather than building a more comprehensive form of institution, ASEAN should focus on strengthening its existing institutional architecture, particularly the Secretariat and its Secretary General. Indeed, the ASEAN Secretariat is its only real live institutional organ, while the rest, consisting of summits, meetings, dialogues, committees, subcommittees and task forces, are periodic events with impermanent offices (Chalermpalanupap 2009: 121). Regarding this matter, one scholar from RSIS asserted that:

*“I would strengthen the coordination role of the Secretariat. I would not reinvent the wheel. I would actually look at the Secretariat and say how can we make this more effective… for instance in terms of implementation of the decision-making process.”*^*10*^

Another scholar from RSIS also had a similar view on this issue:

*“I think the priority is to build up existing institutions rather than creating new ones. For example, Dr Surin (the current Secretary General) needs to be given the power and mandate to initiate policy discussion.”*^*11*^

The former Secretary General also insisted that ASEAN should focus on the existing institutions. He gave an example of one ASEAN institution:

*“The ASEAN Foundation is located in Jakarta. It is not well-capitalized but can be developed into a more substantial body to promote, what we call, the ASEAN Socio-cultural Pillar - identity, belonging can be done under the ASEAN Foundation’s osmosis.”*^*12*^

He also highlighted the difference in political culture between Southeast Asia and Europe:

*“In Europe, having a European court and a European parliament… it seems very easy to do because it has been part of some national cultures. In Southeast Asia, we still very jealously guard our sovereign quality.”*

Taking constructivism into consideration, due to the difference in political culture and regional configurations, I doubt whether the European style of institutionalisation would fully work in ASEAN. Fundamentally, ASEAN has a vast diversity among the members as the political regimes in ASEAN span a wide spectrum ranging from electoral democracy to full-scale authoritarianism, with no genuine liberal democracy having emerged as yet. Thus, to my view, the “copy and paste” idea, or importing the institutional configuration that is based on technocracy and western democracy, does not seem to offer a solution to the problem. However, a number of analysts believe that, in addition to strengthening and empowering the Secretariat, ASEAN should have some new institutional ideas in order to support the overloaded work of the ASEAN Secretariat and increase the Association’s efficiency and effectiveness. For instance, Wanandi (2006: 87) suggested that an ASEAN Consultative Assembly consisting of members of the different parliaments and representatives of civil society could be a fruitful enhancement to the decision-making process, which would make ASEAN more democratic and people-oriented. Further, one Malaysian scholar held the view that ASEAN also needs a research wing and a mechanism that links its institutions to non-state sectors and NGOs. She suggested:

*“One would definitely be a research wing… (Also) I think there is merit in creating some forms of institutional links to the non-state sectors, private actors and NGOs.”*^*13*^

Nevertheless, there are a number of opinions which appear to support a certain degree of supranationalism. Regarding this, Hund (2002: 120) suggested that ASEAN ‘requires centrally managed policies and also more independent and preferably supranational institutions’. In addition, an Indonesian scholar from CSIS showed she agreed with this point of view when she stated:

*“I support (supranationalism). Not in the way that is strong supranational. At least, it needs a body that is for decision-making. In ASEAN, everything is always consensus-based and in a lot of cases, especially sensitive cases like territorial disputes, consensus gets us nowhere.”*^*14*^

To interpret, this does not mean ASEAN should be heading towards a supranational form of integration as has appeared in Europe. In the foreseeable future, ASEAN still has to remain within the current intergovernmental cooperative framework as long as there has been no significant reform to its principal norms and certainly national governments would be reluctant to lose their country’s national rights and control. More realistically, it is rather the budget that is the first thing that needs to be tackled. As Wanandi (2006: 87) proposed, ‘the system now, whereby every member pays the same amount, is no longer realistic. A new formula that is more tenable and could increase the budget adequately should be contemplated’. In my opinion, the system of absolute equal contribution should be reviewed in order to support the excessive tasks of ASEAN institutions, more specifically the ASEAN Secretariat. Despite limiting the funding to its lowest possible level, the current system does not help narrow development gaps between the members and does not reflect the notion of ‘a community of caring and sharing societies’,^15^ something that the association has been trying to promote. However, I would advise that the members’ contributions should perhaps be based on either the possible gain in interest or a country’s ability to pay, that is, the EU’s GDP-based arrangement is one option that should be considered. For example, if each member contributed 1% of its annual GDP to ASEAN, it does not neglect the principle of equality, because all members are asked for 1%, so this is perhaps just a matter of positively rethinking the issue. This is in accord with Emmerson (2007: 438) who opined ‘this step (the GDP-based contribution) would free the Association’s budget from being limited to ten times what the poorest or least supportive member is willing to pay’. I am also convinced that this new formula would help ASEAN to offset the situation whereby there is a clear hierarchy of member’s power and influence. Moreover, I also suggest that ASEAN should be provided with the means to generate its own revenue, for example, through some kind of taxation, import duties or licensing. This would ensure adequate resourcing and financial stability as well as could somehow increase people’s participation and attachment to the association because ASEAN begins to take part in their daily lives.

### Moving forward: the prospects for institutionalisation in ASEAN

Throughout the paper, it has been apparent that having solid, sufficient and efficient forms of institutions is essential for constructing a regional community. Theoretically, constructivism provides a more precise framework to explain ASEAN institutions in that the institutional structure of the organisation can ideationally be viewed as a by-product as well as a representative of its norms and principal values. For according to Stubbs (2008: 455), ‘ideas must be institutionalised in order to be converted into concrete action and have a long lasting impact’. I would hold that the constructivist explanation regarding institutionalisation that surrounding environments, external constraints and international contexts are the key drivers, best fits with what has transpired within ASEAN. That is, the unique configuration of features for Southeast Asia has determined a markedly different institutional path than taken by the EU.

Even though the case of the European Union decisively demonstrates the crucial role of institutions and the importance of supranationalism in the regional integration process, ASEAN has not chosen this path, preferring a non-intrusive manner and placing its faith in cooperating on the basis of intergovernmentalism. The ending of confrontation between Indonesia and Malaysia, the rise of nationalism as a result of colonial experiences and the gaining independence of ASEAN members, the spread of communism, the wars that affected the region and the influence of external powers are the main factors that have shaped this ideational basis of the association. One simple explanation could be that ASEAN was established as an ad hoc resolution in order to meet regional expectations and in response to those perceived challenges. Although the system has responded well to these expectations, to great extent, its current regional architecture has proved insufficient to handle the growing roles and activities as well as to remain in tune with the regional and global trends. In sum, the theoretical explanations, the empirical evidence and the different contexts of regional settings point to the need for a rethink regarding ASEAN’s institutional structure so as to make stronger and thus able to help the association meet the challenges of increased globalisation.

On a practical level, the problems of ASEAN institutions are divided into three major interrelated areas of deficiency. Firstly, its institutions, particularly the ASEAN Secretariat and the Secretary General, lack mandate to ensure compliance or have the ability to initiate policies that can help fulfil ASEAN’s ambition. In general, the ASEAN Secretariat, as a lone central administrative body and the main driving force, has been only tasked with serving and provide administrating support for the national governments without any delegation of power to it. That is, there has not been the intention to build up regional bureaucracies that could promote an independent agenda for integration beyond the scope of intergovernmental cooperation structures (Capannelli and Tan 2012: 14). Instead, supreme power has been wholly retained at the national government level and ASEAN institutions remain at the margins of policy-making. Secondly, the ASEAN Secretariat is currently experiencing financial hardship. This concern has been expressed in much of the literature and by almost all of the interview respondents. This is essentially due to the system of equal member contributions, which has been maintained as low as possible in order to accommodate the less-developed nations, and the dependence on external contributions. Finally, there is strong evidence that ASEAN is understaffed, both in terms of quality and quantity. Moreover, it cannot attract talented and capable people because they do not see “working at the ASEAN Secretariat” as a well-paid and challenging career. These three concerns are clearly interrelated and have become mainstream criticisms of the ASEAN Secretariat that need to be resolved.

For the prospects of institutionalisation in ASEAN, a general conclusion would be that ASEAN institutions should be strengthened and provided with mandate, particularly in terms of policy implementation and compliance, and sufficient financial and human resources to support its administration and activities. In particular, the current contribution system should be substantially revised, because it is not realistic and applicable to the growing activities of the association. Regarding this matter, I would propose that in order to increase financial flexibility and stability the ASEAN Secretariat should also seek other sources of income, rather than solely rely on member contributions and external donors. This could take the form of, for example, import duties, a percentage of tax levied by each member country or even fines from companies that breach ASEAN regulations. In general, I believe that the institutional structure of ASEAN met the task, by and large, of serving the national governments and upholding the association’s norms and therefore there is no need to replicate the EU’s integration experience by a complete overhaul with the imposition of completely new institutional initiatives. As most of the contributors to this research have opined, ASEAN should focus on strengthening its existing institutions by giving them more mandate, more money and more professional staff. However, some of the suggestions about an institutional invention are worth considering and can address deficiencies in the organisation, such as the establishing a research wing and a mechanism that can get non-state actors involved in the process of regional governance. Furthermore, in line with Wah (1998: 165), it is noted that long-term institutional reform cannot be achieved without the reconsideration of the relationships with the wider institutional structure of ASEAN that the Secretariat is nested within, particularly with the Coordinating Council, the Ministerial Bodies, the Committee of Permanent Representatives and the national governments.

Above all, I am convinced that ASEAN decision-makers are aware of the institutional weaknesses in ASEAN and want to address. This is evidenced by the introduction of the ASEAN Charter, which was seen as an important step forward for institutional reform as it strengthened its implementation and dispute-settlement mechanisms as well as consolidating its decision-making structure. However, although the ASEAN Charter did empower the existing ASEAN institutions and provided the Association with a juridical foundation, it seems to have codified traditional norms and practices as well as reinforcing the idea of non-intervention (Narine 2008: 425). Therefore, in the post-Charter era I doubt that ASEAN will become a more effective organisation and all the institutional problems discussed will be properly tackled any time soon. In essence, it would appear to me that all the complications arise from the fact that ASEAN integration does not involve like-minded states in that the members do not share similar political ideologies and values. Furthermore, this political dissimilarity is safeguarded by the association’s norms and the consensus-based decision making, which continue to limit the role of its institutions, thus allowing member countries to enjoy cooperation and exercise power without fear of their sovereignty being at stake. This situation has resulted in deadlock, which has prevented the Association from making any considerable progress and so it ‘remains robustly intergovernmental, with little delegation since it relies on “offshoots” rather than independent agencies to implement policy’ (Capannelli and Tan 2012: 14). All in all, granting mandates and pouring resources into the central institution will not fully resolve the problems, because it would still lack some of the most important ingredients for deeper cooperation. That is, in order to maximum benefits from constructing a regional community, this would need centralised policies, some degrees of supranationalism as well as the national governments’ willingness to cooperate and to delegate powers.

## Endnotes

^1^See the ASEAN Charter, Chapter IV, Article 7–15.

^2^The opinion given in The Jakarta Post, March 07, 2013.

^3^Muhibat 2013. Interviewed by the author [in person] Jakarta, 23 March 2013.

^4^Chalermpalanupap 2012. Interviewed by the author [in person] Singapore, 20 December 2012.

^5^In 2011, ASEAN has a population of approximately 602,658,000.

^6^Yong 2013. Interviewed by the author [in person] Kuala Lumpur, 21 March 2013.

^7^See http://www.euractiv.com/euro-finance/european-top-court-blocks-pay-ri-news-531830

^8^Yong 2013. Interviewed by the author [in person] Kuala Lumpur, 21 March 2013.

^9^Swajaya 2013. Interviewed by the author [in person] Jakarta, 22 March 2013.

^10^Emmers 2013. Interviewed by the author [in person] Singapore, 22 January 2013.

^11^Chong 2012. Interviewed by the author [in person] Singapore, 21 December 2012.

^12^Yong 2013. Interviewed by the author [in person] Kuala Lumpur, 21 March 2013.

^13^Nesadurai 2013. Interviewed by the author [in person] Kuala Lumpur, 5 March 2013.

^14^Muhibat 2013. Interviewed by the author [in person] Jakarta, 23 March 2013.

^15^See the Cebu Declaration Towards One Caring and Sharing Community.

## References

[CR1] Acharya A (2005). Do norms and identity matter? Community and power in Southeast Asia's regional order. Pac Rev.

[CR2] Areethamsirikul S (2010). Can Jakarta become "the Brussels of the East" and the capital of ASEAN.

[CR3] Asian Development Bank (2010). Institutions for Regional Integration: Toward an Asian Economic Community.

[CR4] Beeson M (2008). Institutions of the Asia Pacific: ASEAN, APEC and beyond.

[CR5] Best E (2005). Supranational Institutions and Regional Integration.

[CR6] Bower EZ (2010). Balance & Good Health Come from a Strong Core: Is the ASEAN Secretariat Properly Resourced?.

[CR7] Busse N (1999). Constructivism and Southeast Asian security. Pac Rev.

[CR8] Capannelli G, Tan SS (2012). Institutions for Asian Integration: Innovation and Reform. ADBI Working Paper.

[CR9] Chalermpalanupap T, Emmerson DK (2009). Institutional Reform: One Charter, Three Communities, Many Challenges. Hard choices: security, democracy, and regionalism in Southeast Asia.

[CR10] Chalermpalanupap T (2012). Interviewed by the author [in person] 20 December 2012, Singapore.

[CR11] Chong A (2012). Interviewed by the author [in person] 21 December 2012, Singapore.

[CR12] Cini M (2007). European Union politics.

[CR13] Collins A (2007). Forming a Security Community: Lessons from ASEAN. Int Relat Asia Pac.

[CR14] Desker B (2008). Is the ASEAN Charter Necessary?. RSIS Commentaries No.77/2008.

[CR15] Emmers R (2013). Interviewed by the author [in person] 22 January 2013, Singapore.

[CR16] Emmerson DK (2007). Challenging ASEAN: A "Topological" View. Contemp Southeast Asia.

[CR17] Hernandez C (2007). Institution Building through an ASEAN Charter. Panorama: insights into Southeast Asian and European Affairs.

[CR18] Hund M (2002). From 'neighbourhood watch group' to community? The case of ASEAN institutions and the pooling of sovereignty. Aust J Int Aff.

[CR19] Jetschke A (2009). Institutionalizing ASEAN: celebrating Europe through network governance. Camb Rev Int Aff.

[CR20] Jetschke A, Murray P (2011). Diffusing regional integration: the EU and Southeast Asia. West Eur Polit.

[CR21] Muhibat S (2013). Interviewed by the author [in person] 23 March 2013, Jakarta.

[CR22] Narine S (1998). Institutional theory and Southeast Asia: the case of ASEAN. World Affairs.

[CR23] Narine S (2008). Forty years of ASEAN: a historical review. Pac Rev.

[CR24] Nesadurai H (2012). Chapter prepared for the ADB-funded and ISEAS-coordinated study on "Assessment of Impediments and Actions Required for Achieving an ASEAN Economic Community by 2015". Enhancing the Institutional Framework for AEC Implementation: Designing Institutions that are Effective and Politically Feasible.

[CR25] Nesadurai H (2013). Interviewed by the author [in person] 5 March 2013, Kuala Lumpur.

[CR26] Poole A (2011). Paper presented at The Australian Political Studies Association (APSA) conference. The state versus the Secretariat: Capacity and the norm of equality in ASEAN.

[CR27] Severino R (2009). Regional Institutions in Southeast Asia: The First Movers and their Challenge. Workshop on Evolution of Institutions for Regionalism in Asia and the Pacific.

[CR28] Stubbs R (2008). The ASEAN alternative? Ideas, institutions and the challenge to 'global' governance. Pac Rev.

[CR29] Suryodiningrat M (2009). Defending the defenders - A role for the ASEAN secretary-general.

[CR30] Swajaya N (2013). Interviewed by the author [in person] 22 March 2013, Jakarta.

[CR31] Wah CK, Leong S (1998). ASEAN Institution Building. ASEAN towards 2020: strategic goals and future directions.

[CR32] Wanandi J (2006). ASEAN future challenges and the importance of an ASEAN Charter. ASIEN.

[CR33] Wendt A (1992). Anarchy is what states make of it - the social construction of power-politics. Int Organ.

[CR34] Wendt A (1995). Constructing international-politics. Int Secur.

[CR35] Yong OK (2013). Interviewed by the author [in person] 21 March 2013, Kuala Lumpur.

